# Up-Regulation of Glycogen Synthesis and Degradation Enzyme Level Maintained Myocardial Glycogen in Huddling Brandt’s Voles Under Cool Environments

**DOI:** 10.3389/fphys.2021.593129

**Published:** 2021-03-26

**Authors:** Jin-Hui Xu, Zhe Wang, Jun-Jie Mou, Chuan-Li Wang, Wei-Mei Huang, Hui-Liang Xue, Ming Wu, Lei Chen, Lai-Xiang Xu

**Affiliations:** College of Life Sciences, Qufu Normal University, Qufu, China

**Keywords:** huddling, low temperature, heart, glycogenosomes, glycogen synthetase, glycogen phosphorylase

## Abstract

Small mammals exhibit limited glucose use and glycogen accumulation during hypothermia. Huddling is a highly evolved cooperative behavioral strategy in social mammals, allowing adaptation to environmental cooling. However, it is not clear whether this behavior affects the utilization of glycogen in cold environments. Here, we studied the effects of huddling on myocardial glycogen content in Brandt’s voles (*Lasiopodomys brandtii*) under a mild cold environment (15°C). Results showed that (1) Compared to the control (22°C) group (CON), the number of glycogenosomes more than tripled in the cool separated group (CS) in both males and females; whereas the number of glycogenosomes increased in females but was maintained in males in the cool huddling group (CH). (2) Glycogen synthase (GS) activity in the CS group remained unchanged, whereas glycogen phosphorylase (GYPL) activity decreased, which mediated the accumulation of glycogen content of the CS group. (3) Both GS and GYPL activity increased which may contribute to the stability of glycogen content in CH group. (4) The expression levels of glucose transporters GLUT1 and GLUT4 increased in the CS group, accompanied by an increase in glucose metabolism. These results indicate that the reduced glycogen degradation enzyme level and enhanced glucose transport may lead to an increase in myocardial glycogen content of the separated voles under cool environment; while the up-regulation of glycogen synthesis and degradation enzyme level maintained myocardial glycogen content in the huddling vole.

## Introduction

Low temperature is a stress stimulus for mammals, especially for small mammals as their energy requirements are high due to the large surface area to volume ratio. Moreover, when environmental stressors persist for prolonged periods, small animal tissues and organs are more vulnerable to the impact of external environmental temperature (Gilbert et al., 2010; Wei et al., 2018). Hypothermia can lead to a slowed heart rate, decreased blood flow output, and decreased myocardial contraction and relaxation function (Polderman, 2009; Kelly and Nolan, 2010; Tessier and Storey, 2012;

Chavez et al., 2017). As above, the cardiac muscle of small mammals is more susceptible to low external temperatures. Our previous study showed that, in comparison to warm environmental conditions, Brandt’s voles (*Lasiopodomys brandtii*) under cool (15°C) conditions exhibit myocardial mitochondrial swelling and crista disruption, as well as decreased adenosine triphosphate (ATP) synthase activity (Wang et al., 2020b). Glucose is the energy supply of mitochondria, and thus changes in mitochondrial function may involve changes in glycogen content in tissues (Hall and Mackay, 1933; Tarnopolsky, 2016; Xu et al., 2020). Altered carbohydrate metabolism during hypothermia in mammals is accompanied by abnormalities in glucose metabolism (Baum et al., 1968; Curry and Curry, 1970; Helman et al., 1984). For example, in rats (Popovic, 1960; Fuhrman and Fuhrman, 1963) and rabbits (Bickford and Mottram, 1960), metabolism of both endogenously and exogenously administered glucose is substantially reduced during hypothermia. Furthermore, exposure to only 4 h of cold temperature (15°C) can lead to an increase in myocardial glycogen content in rats (Steffen, 1988), suggesting that the effects of hypothermia on cardiac muscle may involve the balance between glycogen synthesis and degradation.

Glycogen is a branched polymer of glucose and stores energy in times of nutritional sufficiency for utilization in times of need. Glycogen synthase (GS), a key enzyme for synthesis, polymerizes UDP-glucose to form glycogen granules, with phosphorylated GS (P-GS) being its active state (Palm et al., 2013; Zeqiraj and Sicheri, 2015; Wang et al., 2019). Glycogen phosphorylase (GYPL) is a rate-limiting enzyme that breaks down glycogen granules to glucose (Agius, 2010; Mavrokefalos et al., 2015). The direct pathway of glycogen synthesis requires the transport of glucose into cells by one or several glucose transporters (GLUTs) (Thorens and Mueckler, 2010). GLUT1 is widely distributed and provides basal glucose transport; GLUT4 is up-regulated by insulin and is important in insulin-sensitive tissues, such as skeletal muscle and adipose tissue; and GLUT2 is prominent in the liver and β-cells of the pancreas and admits glucose based on a positive glucose gradient between blood and tissue (Roach et al., 2012). Research on hibernating Daurian ground squirrels (*Spermophilus dauricus*) has shown that the increase in glycogen content in skeletal muscle in winter is mainly due to the maintenance of P-GS and decrease in GYPL protein expression (Wang et al., 2019). Thus, studies on the above factors could help reveal the mechanism related to changes in myocardial glycogen content under cool environments.

Huddling is a social thermoregulatory behavior, defined as the active aggregation of nestled animals. It is a cooperative group behavior, permitting individuals involved in social thermoregulation to minimize heat loss and thereby lower energy expenditure, possibly allowing reallocation of saved energy to other functions (Gilbert et al., 2010; Douglas et al., 2017). It is commonly exhibited in small mammals and birds to reduce heat and energy loss under cold environments (Jefimow et al., 2011; Wojciechowski et al., 2011; Sukhchuluun et al., 2018; Zhang et al., 2018). Research has shown that many mammals, such as degu (*Octodon degus*), Damaraland mole-rat (*Cryptomys damarensis*), and Natal mole-rat (*C. hottentotus natalensis*), huddle when the ambient temperature is lower than 15–20°C, with an energy saving of up to 30% (Kotze et al., 2008; Nunez-Villegas et al., 2014). Research on Eastern pygmy possums (*Cercartetus nanus*) has shown that huddling in mild low temperatures (14°C) can reduce energy consumption by up to 50% (Namekata and Geiser, 2009). The benefits of huddling in energy conservation (Scantlebury et al., 2006; Kotze et al., 2008), local environmental heating (Nowack and Geiser, 2016), and survival (Sealander, 1952) have also been studied in several species. Overall, huddling individuals exhibit increased survival, lower food intake, decreased body mass loss, increased growth rate, more constant body temperature, and reduced metabolic rate (Gilbert et al., 2010). To date, previous studies have primarily focused on morphological and physiological changes in animal bodies under various temperatures. However, no studies have reported on changes in myocardial glycogen in mammals under different temperatures.

Brandt’s voles are small non-hibernating herbivorous rodents widely distributed among the Inner Mongolian grasslands of Northern China, dry steppe zone of Mongolia, and southeast Baikal region of Russia. They are highly socialized animals that huddle in winter as an adaptation to their harsh habitats (Zhang et al., 2018), which differs substantially from model animals. Research has shown that mild cooling can significantly change the morphology of mitochondria in the cardiac muscle of Brandt’s voles (Xu et al., 2019; Wang et al., 2020a). Furthermore, their metabolic rate and thermogenic capacity decrease but activity increases compared with separated individuals under low temperatures, suggesting that huddling is a good strategy for small mammals to cope with cold environments (Sukhchuluun et al., 2018). Glycogen is one of the most important energy supply substances in muscles. However, the role of myocardial glycogen in adaptive huddling has not yet been reported. Therefore, we hypothesized that a cool environment could cause an increase in myocardial glycogen content in Brandt’s voles. We also hypothesized that huddling could effectively alleviate this change. To test these hypotheses, we observed the ultrastructure of cardiac muscle in huddling and individual (separated) Brandt’s voles under mild temperature differences (normal: 22°C; cool: 15°C) in autumn. We also determined the protein expression levels of glucose transport glycogen synthesis, and glycogen degradation-related signals. We further explored the underlying molecular mechanism related to the effects of a mild cold environment and huddling on changes in myocardial glycogen content.

## Materials and Methods

### Ethics Statement

All procedures followed the Laboratory Animal Guidelines for the Ethical Review of Animal Welfare (GB/T 35892-2018) and were approved by the Animal Care and Use Committee of Qufu Normal University (Permit Number: dwsc 2019012).

### Animals and Groups

Forty-eight adult voles were captured and housed as described previously (Wang et al., 2020b). The voles were acclimated to laboratory conditions for 2 weeks. They were housed four animals per cage (28 × 18 × 12 cm) at an ambient temperature of 22 ± 2°C, relative humidity of 55 ± 5%, and light/dark regime of 12 h:12 h (light on from 06:00 to 18:00). Food (standard rabbit chow, Pengyue Experimental Animal Breeding Co., Ltd., China) and water was provided *ad libitum* and wood shavings were used as bedding. Based on body weight, a total of 24 males (28–50 g, average 38 g) and 24 female (27–54 g, average 33 g) adult voles were randomly divided into three groups, respectively. Control group (CON): Voles were continuously housed under an ambient temperature of 22 ± 2°C, with four animals in each cage (two males and two females), similar to their normal state in autumn. Cool huddling group (CH): Voles were housed together in a cage (two males and two females) under an ambient temperature of 15°C. The group size (four voles in each cage) ensured most animals remained inactive in a huddle (Sukhchuluun et al., 2018). Cool separated group (CS): Voles were housed individually in cages at an ambient temperature of 15°C. The three treatment groups were maintained under the same relative humidity (55 ± 5%) and light regime (12 h: 12 h light/dark, light on from 06:00 to 18:00). Animal treatment started in late September and lasted 8 weeks (Wang et al., 2020b).

### Sample Preparation

All animals were sacrificed by CO_2_ asphyxiation between 08:00 and 11:00 a.m. on the last day of the experiment (Sukhchuluun et al., 2018; Wang et al., 2020b). After the rapid removal of cardiac muscle, portions of the ventricles were immediately excised and fixed in glutaraldehyde. Specimens were fixed in 1% osmium tetroxide in the same buffer, dehydrated with a graded series of ethanol, and embedded in epoxy resin. The remaining cardiac muscle was frozen in liquid nitrogen and stored at −80°C. All procedures were carried out in accordance with the approved guidelines.

### Transmission Electron Microscopy (TEM)

The cardiac muscle samples were cut into blocks and immersed in 3% glutaraldehyde-paraformaldehyde. The blocks were then dehydrated in a graded series of ethanol and embedded in epoxy resin, with TEM then performed as described previously (Wang et al., 2020a). Semi-thin sections of the tissue samples were stained with methylene blue (Biazik et al., 2015), then adjusted under the microscope and sliced with an ultramicrotome (LKB-NOVA, United States). The ultrathin sections were double stained with Reynolds’ lead citrate and ethanolic uranyl acetate (Reynolds, 1963) and then examined via TEM (Hitachi, HT7800, Japan). Images were processed with NIH Image-Pro Plus 6.0. Images were analyzed using the measurement tools provided by the software. Glycogenosome densities were determined within a defined region (4 μm^2^ area) at a minimum of three locations within an image taken at 25,000 × magnification.

### GS and GYPL Activity

Samples stored at −80°C were used to detect GS and GYPL activity. GS activity was determined by measuring the rate of NADH decline at 450 nm using a Glycogen Synthase Assay Kit (20E10Y14, Shanghai Hengyuan Biological Technology Co., Ltd., China) according to the manufacturer’s instructions (Ouyang et al., 2018). GYPL activity was determined by measuring the rate of NADPH increase at 450 nm with a Glycogen Phosphorylase Activity Assay Kit (20H10L15, Shanghai Hengyuan Biological Technology Co., Ltd., China) according to the manufacturer’s instructions (Song et al., 2018).

### Glycogen Quantification

Samples stored at −80°C were used to detect glycogen content. The amount of glycogen in the myocardia from the three groups was determined with a Glycogen Assay Kit (BC0340, Solarbio, Beijing, China). Glycogen levels were normalized by cell protein concentration measured using the BCA assay (Zhao et al., 2017).

### Western Blotting

Total protein was extracted from the tissues and solubilized in sample buffer (100 mM Tris, pH 6.8, 5% 2-β-mercaptoethanol, 5% glycerol, 4% SDS, and bromophenol blue), with the extracts of cardiac protein then resolved via SDS-PAGE [10% Laemmli gel with an acrylamide/bisacrylamide ratio of 29:1 and 98% 2,2,2-trichloroethanol (Aladdin, JI522028, China)]. To study protein expression in different tissues, we used total protein content as a reference. After electrophoresis, the gel was irradiated on the UV platform of the electrophoresis gel imaging analysis system (Bio-Rad, California, United States) for 5 min, with the signal then collected. As described previously (Li and Shen, 2013; Posch et al., 2013), the original image captured with no gain was stored. The fluorescence intensity of each lane (after removal of background fluorescence intensity) was determined with Image-Pro Plus 6.0, which contains an internal reference to correct the fluorescence intensity of the target protein. The proteins were then electrically transferred to polyvinylidene fluoride (PVDF) membranes (0.45 μm pore size) using a Bio-Rad wet transfer apparatus. The blotted membranes were blocked with 5% skimmed milk powder in Tris-buffered saline (TBS; 150 mM NaCl, 50 mM Tris-HCl, pH 7.5) and incubated with rabbit anti-glycogen phosphorylase (1:1,000, #ab198268, Abcam, Cambridge, United Kingdom), rabbit anti-glycogen synthase (1:1,000, #3886, Cell Signaling Technology CST, Danvers, MA, United States), rabbit anti-phospho glycogen synthase (1:1,000, #3891, CST), rabbit anti-glucose transporter type 1 (1:500, #21829, Proteintech, China), rabbit anti-glucose transporter type 2 (1:500, #20436, Proteintech, China), and rabbit anti-glucose transporter type 4 (1:500, #21048, Proteintech, China) in TBS containing 0.1% BSA at 4°C overnight. The membranes were then incubated with IRDye 800 CW goat anti-rabbit secondary antibodies (1:5,000, #31460, Thermo Fisher Scientific, Rockford, IL, United States) for 90 min at room temperature and visualized with an Odyssey scanner (Bio-Rad, CA, United States). Quantification of blots was performed using NIH Image-Pro Plus 6.0.

### Statistical Analyses

The normality of data and homogeneity of variance were tested by Shapiro-Wilk and Levene tests, respectively. All data exhibited normal distribution and homogeneous variance. Double-factor variance analysis [two-way analysis of variance (ANOVA)] was used to compare differences between treatment and sex. Results were significant at *P* < 0.05. Data are expressed as mean ± standard deviation (Mean ± SD). All statistical analyses were conducted using SPSS 19.0.

## Results

### Ultrastructural Changes in Number of Glycogenosomes

**FIGURE 1:**
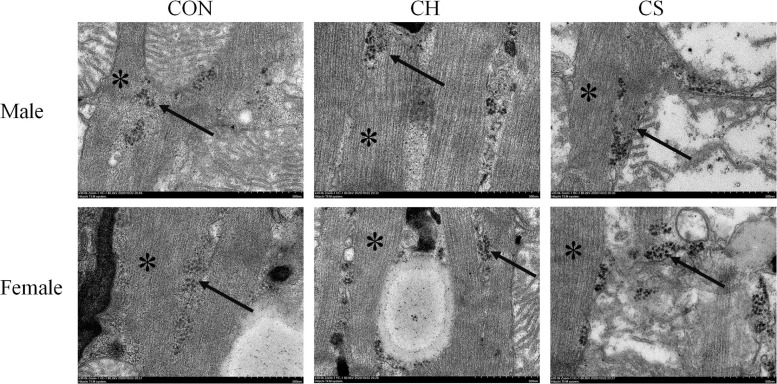
Ultrastructural distribution of myocardial glycogenosomes in Brandt’s voles. Arrow indicates glycogenosome. Muscle filaments (see asterisk) was well arranged. Scale bar = 0.5 μm.

Glycogenosome clusters were observed, with each glycogenosome showing a diameter of ∼30 nm. Most glycogenosomes were distributed between the muscle filaments, with a small number distributed around the mitochondria (Figure 1).

**FIGURE 2:**
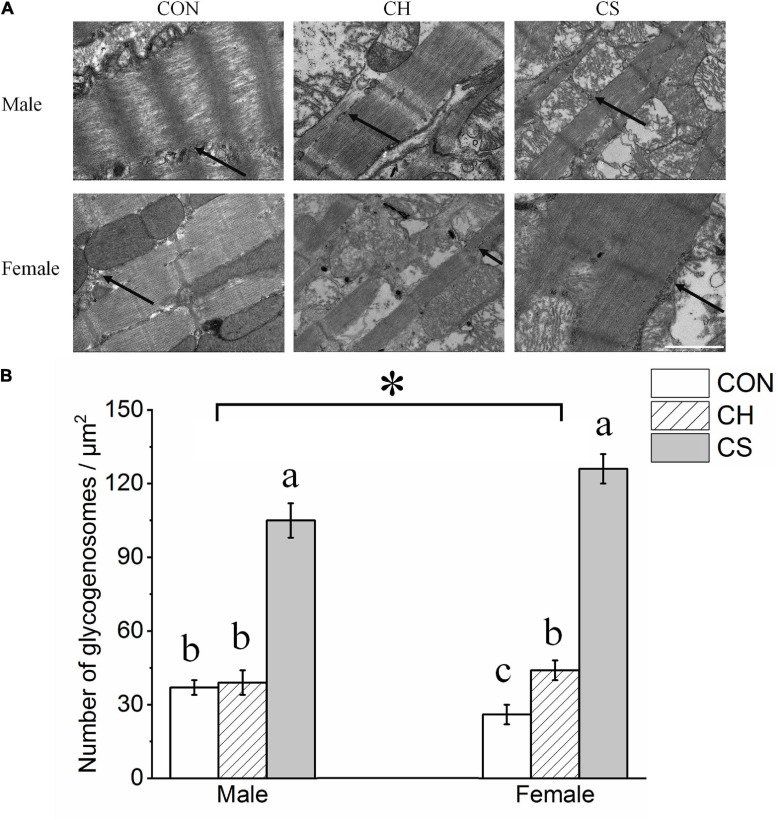
Changes in number of myocardial glycogenosomes in Brandt’s voles. **(A)** Myocardial glycogenosomes in three treatment groups. Arrow indicates glycogenosome. Scale bar = 1 μm. **(B)** Bar graph depicting changes in number of glycogenosomes. Values are mean ± SD. Six figures were analyzed in each sample; eight samples were analyzed in each group. CON, control group; CH, cool huddling group; CS, cool separated group. Different letters identify statistically significant differences among temperature treatment groups (*P* < 0.05). *P < 0.05 significant differences between males and females.

Figure 2A shows the distribution of glycogenosomes at low magnification. In the CS group, the number of glycogenosomes was more than triple that in the CON and CH groups (*P* < 0.05). In addition, the number was significantly higher (*P* < 0.05) in females than in males (Figure 2B).

### Glycogen Quantification

**FIGURE 3:**
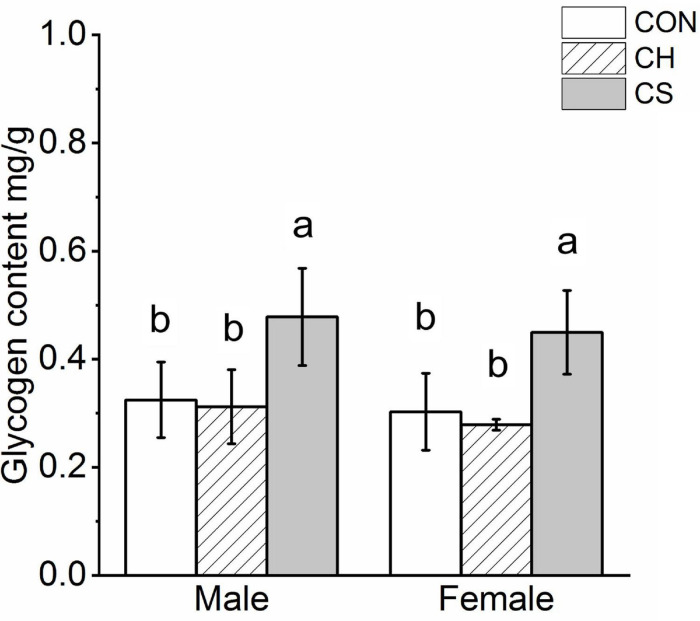
Changes in content of myocardial glycogenosomes in Brandt’s voles. Glycogen levels were normalized by cell protein concentration measured using the BCA assay. Different letters identify statistically significant differences among temperature treatment groups (*P* < 0.05). CON, control group; CH, cool huddling group; CS, cool separated group.

Glycogen quantification showed significant accumulation in the CS group (*P* < 0.05), but no significant differences were observed between the CON and CH groups in either males or females (Figure 3).

### Changes in GS and GYPL

**FIGURE 4:**
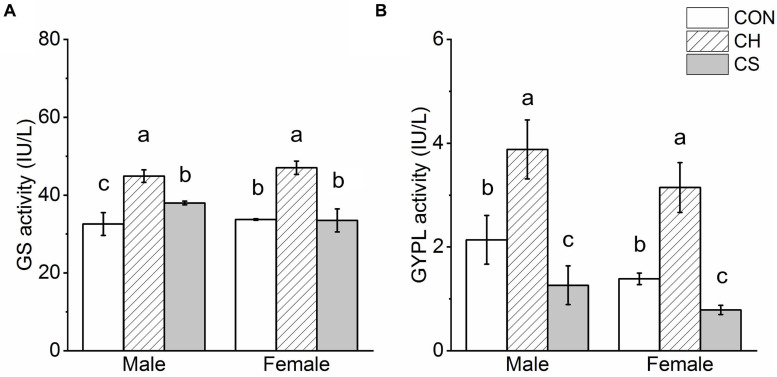
Glycogen synthase (GS) and glycogen phosphorylase (GYPL) activity in cardiac muscle of voles. **(A)** GS activity. **(B)** GYPL activity. Values are mean ± SD. *n* = 8. CON, control group; CH, cool huddling group; CS, cool separated group. Different letters indicate significant differences among temperature treatment groups (*P* < 0.05).

Results showed that GS activity in the CH group was significantly higher than that in the CON and CS groups (*P* < 0.05), but there were no significant differences between the CON and CS group in females. Furthermore, among the three groups, GYPL activity was highest in the CH group (*P* < 0.05) and lowest in the CS group (*P* < 0.05) (Figure 4).

### Changes in Protein Expression of Glycogen Synthesis-Related Proteins

**FIGURE 5:**
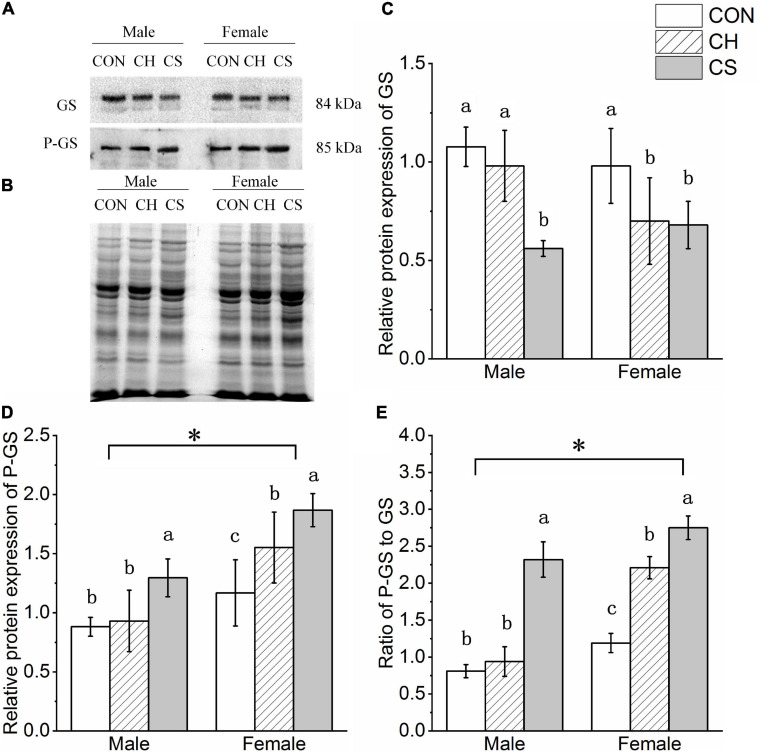
Changes in protein expression levels of glycogen synthesis-related factors in cardiac muscle of Brandt’s voles. (A) Representative immunoblots of GS and P-GS in cardiac muscle. **(B)** Representative polyacrylamide gel of total protein. **(C)** Relative protein expression of GS. **(D)** Relative protein expression of P-GS. **(E)** Ratio of P-GS to GS. Values are mean ± SD. *n* = 8. CON, control group; CH, cool huddling group; CS, cool separated group. Different letters identify statistically significant differences among temperature treatment groups (*P* < 0.05). ^∗^*P* < 0.05 significant differences between males and females.

The GS and P-GS concentrations were detected by western blot analysis, as shown in Figure 5. Representative polyacrylamide gels of total protein are shown in Figure 5B.

The relative protein expression levels of GS and P-GS showed different trends among the three treatment groups. Specifically, the protein expression levels of GS in the CS group were lower than the levels in the CH and CON groups, whereas protein expression levels of P-GS in the CH and CS groups were higher than levels in the CON group (*P* < 0.05). Levels of P-GS was higher (*P* < 0.05) in females than in males (Figures 5C,D).

The P-GS to GS ratio is one of the most direct indicators of glycogen synthesis. Here, the ratio trend among the three treatment groups was CON < CH < CS (*P* < 0.05). The ratio was also higher (*P* < 0.05) in females than in males (Figure 5E).

### Changes in Protein Expression of Glycogen Decomposition-Related Proteins

**FIGURE 6:**
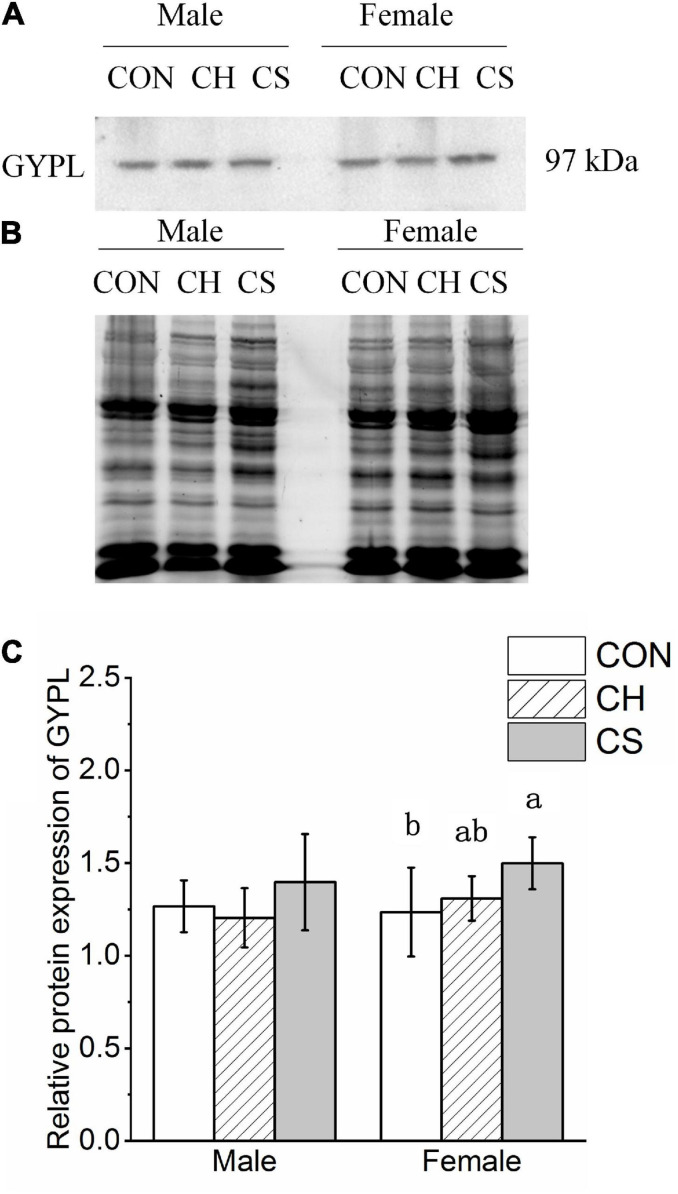
Changes in protein expression levels of glycogen degradation-related factors in cardiac muscle of Brandt’s voles. **(A)** Representative immunoblots of GYPL in cardiac muscle. **(B)** Representative polyacrylamide gel of total protein. **(C)** Relative protein expression of GYPL. Values are mean ± SD. *n* = 8. CON, control group; CH, cool huddling group; CS, cool separated group. Different letters identify statistically significant differences among temperature treatment groups (*P* < 0.05).

The content of GYPL was detected by western blot analysis, as shown in Figure 6. Representative polyacrylamide gels of total protein are shown in Figure 6B.

The relative protein expression of GYPL showed a slight change among the three treatment groups. Specifically, levels were higher in CS group females than in CON group females (Figure 6C).

### Changes in Protein Expression of Glucose Transporter Proteins

**FIGURE 7:**
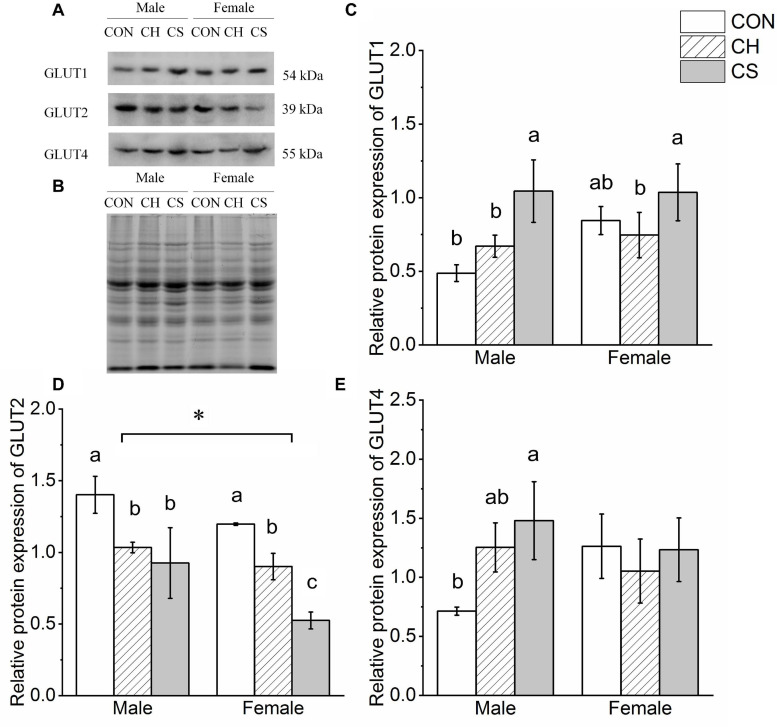
Changes in protein expression levels of glucose transporter proteins in cardiac muscle of Brandt’s voles. **(A)** Representative immunoblots of GLUT1, GLUT2, and GLUT4 in cardiac muscle. **(B)** Representative polyacrylamide gel of total protein. **(C)** Relative protein expression of GLUT1. **(D)** Relative protein expression of GLUT2. **(E)** Relative protein expression of GLUT4. Values are mean ± SD. *n* = 8. CON, control group; CH, cool huddling group; CS, cool separated group. Different letters identify statistically significant differences among temperature treatment groups (*P* < 0.05).

The contents of GLUT1, GLUT2, and GLUT4 were detected by western blot analysis, as shown in Figure 7. Representative polyacrylamide gels of total protein are shown in Figure 7B.

The relative protein expression of GLUT1 increased in the CS group compared with the other groups in both males and females (*P* < 0.05). The relative protein expression of GLUT2 showed the same trend, i.e., CON > CH > CS, but there was a significant difference between males and females. The relative protein expression of GLUT4 in males was markedly higher in the CS group than in the CON group (*P* < 0.05), but there were no differences in females among the three groups.

## Discussion

We studied the effects of a cool environment on the number of cardiac glycogenosomes and glycogen content in huddling Brandt’s voles, as well as the underlying mechanism related to the regulation of glycogenosome number. One of the most important findings of this study is the ultrastructural observation of a significant increase in the number of cardiac glycogenosomes in the CS group, as verified by the glycogen content results.

Changes in myocardial glycogen in mammals during long-term cool exposure have not been reported previously, although our results are consistent with those of myocardium under short-term hypothermia and skeletal muscle under long-term hypothermia, as the major types of muscle fibers in ventricles are similar to those in soleus muscle (Schaub et al., 1989). Research on rats has shown that glycogen content in the myocardium is significantly increased after only 4 h of exposure to 15°C (Steffen, 1988). Furthermore, Daurian ground squirrels experience an increase in glycogen concentration in the soleus muscle after 2 months of low temperature exposure in winter (Wang et al., 2019). Excessive glycogen accumulation in the heart can lead to degenerative changes such as arrhythmia, cardiac hypertrophy, and hypotonia (Kanungo et al., 2018). In this study, glycogen content in the myocardium of the CS group was significantly higher than that of the CON group. This indicates that hypothermia may cause significant degenerative damage to the myocardium of small mammals and may involve disrupting the balance between glycogen synthesis and decomposition. In addition, our previous study indicated that ATP synthase activity in the myocardial mitochondria of Brandt’s voles under cool conditions is significantly lower than that observed under warm environments, which may lead to a decrease in glucose utilization in the mitochondria (Wang et al., 2020b). Thus, this may be one of the reasons for glycogen accumulation in the CS group.

Here, compared with the CON group, GS activity in the myocardium increased in the CS group males but remained stable in the CS group females, indicating that the level of glycogen synthesis did not decrease. In addition, in the CS group, GYPL activity decreased in the myocardium of both males and females, indicating that glycogen decomposition was weakened. Therefore, the maintenance of glycogen synthesis enzyme and reduction of glycogen degradation enzyme in the CS group may be one of the main reasons for the increase in glycogen content/glycogen particle accumulation in the myocardium. One thing to note is that the expression of GS protein was significantly decreased in the CS group, but its phosphorylation rate, the active state of GS (Greenberg et al., 2006) was significantly increased, which may be a major mechanism related to the unchanged enzyme activity level of GS.

Surprisingly, compared with the CON group, the content of glycogen in the myocardium of the CH group remained unchanged, with the synchronous increase in glycogen synthesis and degradation enzyme likely responsible for the maintenance of glycogen stability. This suggests that the effect of low temperature on glycogen synthesis enzyme can be significantly alleviated by huddling behavior. Here, huddling behavior completely or partially alleviated the increase in glycogen content caused by the decrease in glycogen degradation enzyme in the myocardium of voles following cold environment exposure by increasing glycogen decomposition. Normal glycogen metabolism is the basis of exercise in mammals (Consitt et al., 2019; Moniz et al., 2020). Earlier studies on Brandt’s voles showed that activity is higher in huddling groups than separated groups under cool environments (Sukhchuluun et al., 2018). Thus, we speculated that the similar level of glycogen metabolism in the myocardium of the CH group and CON group compared to that in the CS group may be the one of the underlying reasons.

Glycogen synthesis and decomposition also depend on changes in glucose metabolism (Chen and Phelix, 2019). In this study, the protein expression levels of glucose transporters GLUT1 and GLUT4 in the CS group males were significantly higher than in the CON group males, which may contribute to intracellular glucose accumulation and glycogen content increase. In female voles, the protein expression of GLUT1 was significantly higher in the CS group than in the CH group, which may be one of the reasons why glycogen content in the myocardium of the CS group was higher than that of the CH group.

In summary, we explored the regulatory mechanism related to the balance between glycogen synthesis and degradation on the number in myocardial glycogenosomes of huddling and separated Brandt’s voles under cool environments. Results showed that a cool environment led to an increase in myocardial glycogen content in voles, which could be alleviated by huddling behavior, and may be a good consequence of the collective overwintering behavior of socialized animals. The activity of glycogen phosphorylase decreased, and the protein expression of GLUT1 and GLUT2 increased in CS group, indicating that the glycogen degradation enzyme decreased and glucose transport increased in the CS group. The activities of glycogen synthase and glycogen phosphorylase increased in the CH group, suggesting that the synthesis and decomposition of glycogen were increased in the CH group. These results indicate that the reduced glycogen degradation enzyme level and enhanced glucose transport may lead to an increase in myocardial glycogen content in the separated voles under cool environment; while the up-regulation of glycogen synthesis and degradation enzyme level maintained myocardial glycogen content in the huddling voles.

## Data Availability Statement

The original contributions presented in the study are included in the article/Supplementary Material, further inquiries can be directed to the corresponding author/s.

## Ethics Statement

All procedures followed the Laboratory Animal Guidelines for the Ethical Review of Animal Welfare (GB/T 35892-2018) and were approved by the Animal Care and Use Committee of Qufu Normal University (Permit Number: dwsc 2019012).

## Author Contributions

J-HX and ZW conceived and designed the research, edited the manuscript, approved the final version, and drafted the manuscript. J-HX, ZW, J-JM, C-LW, and W-MH performed the experiments. J-JM and ZW analyzed the data and prepared the figures. J-JM interpreted the experimental results. H-LX, MW, LC, and L-XX provided experimental guidance and suggestions for revision. All authors contributed to the article and approved the submitted version.

## Conflict of Interest

The authors declare that the research was conducted in the absence of any commercial or financial relationships that could be construed as a potential conflict of interest.
