# Supplementary material for: Up-Regulation of Glycogen Synthesis and Degradation Enzyme Level Maintained Myocardial Glycogen in Huddling Brandt’s Voles Under Cool Environments
Source: Front Physiol. 2021 Mar 26;12:593129. doi: 10.3389/fphys.2021.593129 (PMC8033036; doi:10.3389/fphys.2021.593129)

Original figures of western blot

Original figures of GS western blot in Fig 5

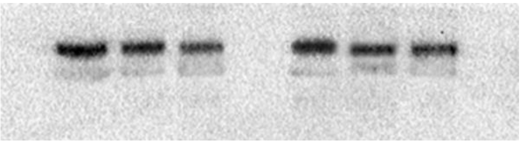

Original figures of P-GS western blot in Fig 5

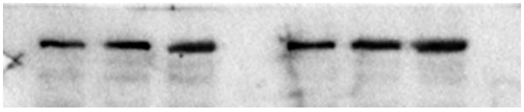

Original figures of PYGL western blot in Fig 6

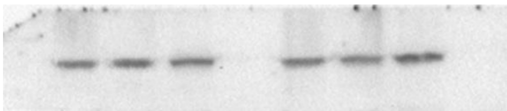

Original figures of GLUT1 western blot in Fig 7

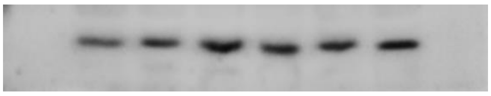

Original figures of GLUT2 western blot in Fig 7

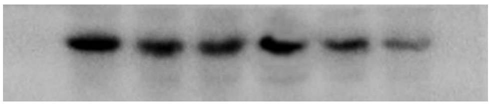

Original figures of GLUT4 western blot in Fig 7

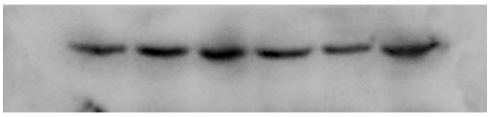

Original figures of total protein western blot in Fig 5

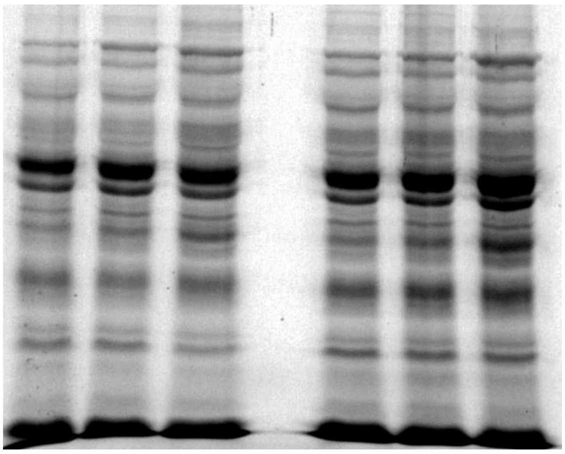

Original figures of total protein western blot in Fig 6

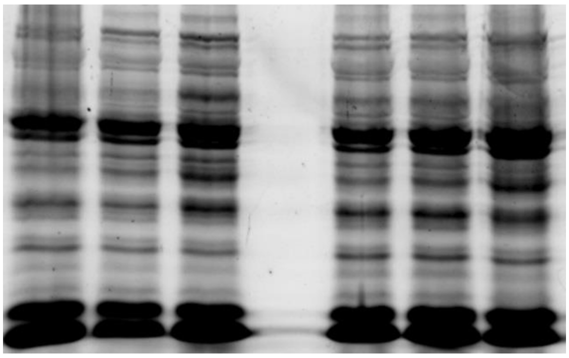

Original figures of total protein western blot in Fig 7

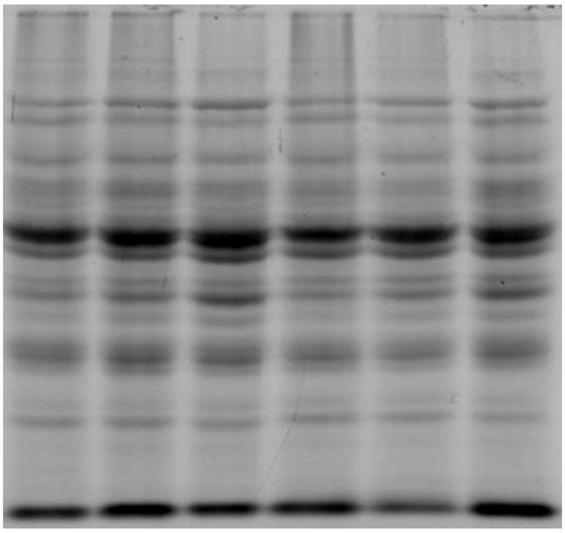

The picture of Brand's voles huddling together in the CH group.

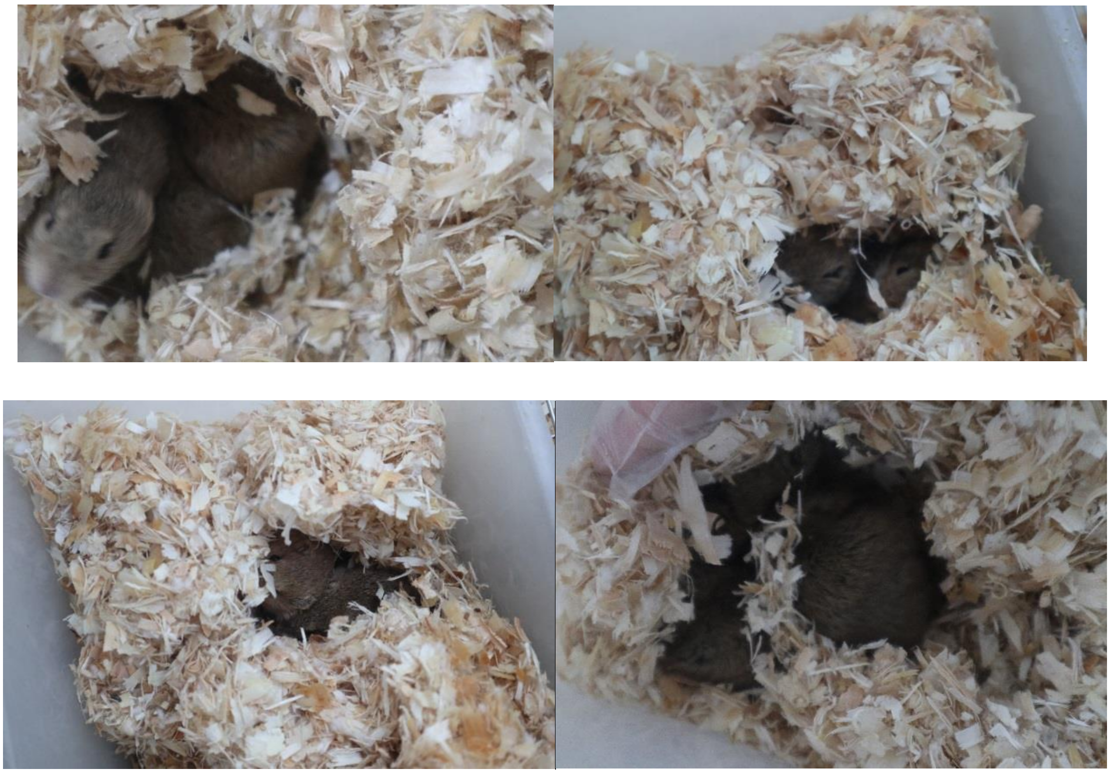

Supplement: Supplementary file 1 [file Image_1.pdf]
